# Structural, thermal, and transport properties of La_0.67_Sr_0.33_MnO_3_ nanoparticles synthesized *via* the sol–gel auto-combustion technique

**DOI:** 10.1039/c7ra09883a

**Published:** 2018-01-04

**Authors:** M. Saleem, Dinesh Varshney

**Affiliations:** Materials Science Laboratory, School of Physics, Devi Ahilya University Vigyan Bhavan, Khandwa Road Campus Indore 452001 India vdinesh33@rediffmail.com +91-731-2467028 +91-731-2467028

## Abstract

Herein, rare-earth manganite, La_0.67_Sr_0.33_MnO_3_, has been prepared by a citric acid-assisted sol–gel auto-combustion method at a maintained pH value of 11. Room-temperature X-ray diffraction (RT-XRD) data analysis revealed a rhombohedral structure for the sample with the space group *R*3*c*, which was further confirmed by synchrotron radiation X-ray diffraction (SR-XRD). Rietveld refinement was carried out for both spectra, which confirmed the SR-XRD and RT-XRD results and the various structural parameters. To determine any of the phase transitions in the sample, temperature-dependent X-ray diffraction corresponding to the temperatures of 100 K, 200 K, 250 K, and 325 K was carried out, and no new phase was found. Temperature-dependent Raman characterization confirmed the metallic phase of the sample with the reduced Jahn–Teller distortion. Scanning electron microscopy confirmed the growth in the grain size as a result of a high sintering temperature. Compositional verification was conducted using energy-dispersive analysis of X-ray diffraction (EDAX). Low-temperature dc resistivity measurement showed a metal-insulator transition temperature (*T*_MI_) of ≈178 K. The DSC-specific heat measurement shows the ferromagnetic metallic nature where heat capacity increases with an increase in temperature.

## Abstract 2

Herein, rare-earth manganite, La_0.67_Sr_0.33_MnO_3_, has been prepared by a citric acid-assisted sol–gel auto-combustion method at a maintained pH value of 11.

## Graphical abstract

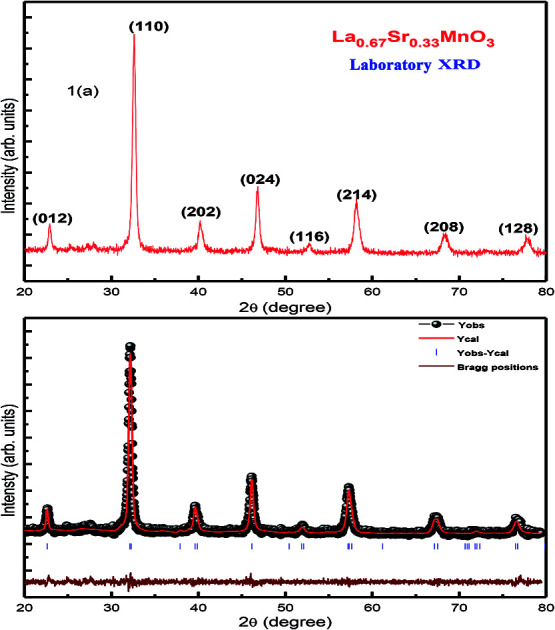

## 1. Introduction

Rare-earth manganites of perovskite structure with the general chemical formula Ln_1−*x*_A_*x*_MnO_3_ (Ln^3+^ is a lanthanide ion and A^2+^ is an alkaline-earth ion) have attracted the core concentration of scientists for more than two decades. By virtue of the close correlation between orbital, charge, spin, and lattice degrees of freedom and the associated diversity of physical properties, these compounds have attracted the keen interest of the scientific community.^1–3^ The modified manganites put forward a good way to explore the properties of strongly correlated electron systems. The physical properties of manganites are highly sensitive to the preparation method, type of unit cell symmetry, size effects, concentration of a dopant, non-stoichiometry, and presence of ions with different valence states at suitable crystallographic sites.^4–6^

Theoretical and experimental results indicated a close correlation between structural, orbital, and electronic degrees of freedom that must be elaborated to explore the complex physics underlying the unique properties of rare-earth manganites. The parent compound LaMnO_3_ exhibiting an orthorhombic structure in particular is considered as the prototype of a coherent Jahn–Teller (JT) system accompanied by cooperative tetragonal deformation of the MnO_6_ octahedra.^7^ Its structure is a sequence of alternate short and long Mn–O bonds in the *ab* plane. Existence of a d-type orbital-ordered state that plays an important role in stabilizing the anisotropic A-type antiferromagnetic state has been explained.^8–10^

The replacement of a trivalent La with a divalent (Ca and Sr) ion corresponds to an effective hole doping. In the 0.2 < *x* < 0.5 concentration range, colossal magnetoresistance and a ferromagnetic metallic ground state are observed, which are qualitatively well explained *via* the double exchange (DE) mechanism.^6,11^ Magnetoresistance (MR) is a special property of doped manganites as it provides favourable conditions for the practical application of manganites. Although the need of the hour is materials with high-room-temperature MR at relatively low magnetic fields, the present curiosity about manganites is to seek the physics of strongly correlated systems, particularly the correlation among orbital, charge, spin, and lattice degrees of freedom.^12,13^

The parent compound LaMnO_3_ is an anti-ferromagnetic semiconductor with the magnetic structure of A-type. The weak ferromagnetic component is attributed to the anti-symmetric exchange. The Neel temperature of LaMnO_3_ is about 140 K. The magnetic properties of manganites are related to the spin of manganese ions because their orbital magnetic moments are frozen into the crystalline field of anions, whereas La^3+^ and O^2−^ ions are diamagnetic.^14,15^

La_0.67_Sr_0.33_MnO_3_ (LSMO) belonging to the hole-doped manganite family is known to be a potential candidate for technological applications as it has a ferromagnetic transition temperature (*T*_C_) around 380 K and a large magnetic moment at room temperature.^16^ This colossal magnetoresistive (CMR) material shows the ground states of a spin-canted insulator, ferromagnetic (FM) insulator, ferromagnetic metal (FMM), antiferromagnetic (AFM) insulator, and antiferromagnetic metal, whereas paramagnetic insulator and metallic behaviours are observed at high temperatures for different Sr doping concentrations.^17^

The sol–gel auto-combustion method is a versatile solution technique used to obtain ultrafine, homogenous powders of a variety of glass and ceramic materials at low temperatures in a short span of time. This method is widely and successfully used for the synthesis of metal oxides at relatively low processing temperatures, free from foreign ions with precise control of the doping level and the particles in the nano-size range. The sol–gel technique has many advantages, such as a large surface area that will enhance the sensing properties apart from simple and low-cost processing, ability to coat large and complex shapes, a porous structure desirable for gas sensor application, and a remarkable possibility to control the particle size, over other methods.

Keeping in mind the abovementioned features of the sol–gel auto-combustion technique, the present study has been carried out to investigate the structure of the LSMO for phase purity using different structure probing techniques; in addition, the best advantage of the present study is the preparation with the maintenance of pH by the addition of ammonia to enhance cation binding to citrate as well as the homogeneity and stability of metal citrate solutions. It also prevents precipitation of individual hydroxides. Citric acid is used as a chelating agent for metal ions and as an organic fuel during the calcination process, and ethylene glycol is used because of its strong reducing power and relatively high boiling point (∼197 °C) to control the particle size. Influence of ammonia, citric acid, and glycol on the various physical properties of the material has been studied.

## 2. Experimental details

The La_0.67_Sr_0.33_MnO_3_ sample was synthesized by the sol–gel auto-combustion (SGAC) method. Stoichiometric amounts of high-purity lanthanum nitrate [La(NO_3_)_3_·6H_2_O], anhydrous strontium nitrate [Sr(NO_3_)_2_], and manganese nitrate [Mn(NO_3_)_2_·6H_2_O] were dissolved in distilled water under continuous stirring, and then, citric acid and glycol were added to make a metal complex while maintaining the pH value at 11. When all the reactants completely dissolved, the solution was mixed and heated at 80 °C; this resulted in the formation of the gel. The gel was dried and calcined at 800 °C for 6 h to remove the remaining organic materials and decompose the nitrates of the gel. The resulting powder was pressed into pellets and sintered for 10 h at 1150 °C.

The crystal structure, type of phase, and crystallite size of the La_0.67_Sr_0.33_MnO_3_ nanopowder were identified *via* the X-ray powder diffraction technique at room temperature using a Bruker D8-Advance X-ray diffractometer with CuKα_1_ (1.5406 Å) radiation. The data were obtained with a step size of 0.02° over the angular range 2*θ* (20° < 2*θ* < 80°) by generating X-ray by 40 kV and 40 mA power settings. Rietveld refinement was conducted on the XRD data using the Fullprof refinement software.^18^ The sample was further subjected to synchrotron radiation XRD to investigate its spectrum and hence the structure using the angle-dispersive X-ray diffraction (AD-XRD) beam line (BL-12) at the Indus-2 synchrotron source using an X-ray of wavelength ≈ 0.8042 Å. To verify the presence of any other phase or any phase transition, low-temperature X-ray diffraction (LTXRD) was performed in the temperature range from 100 K to above room temperature, and the data with a step size of 0.02° was obtained over the angular range 2*θ* (10° < 2*θ* < 110°).

Raman characterization was carried out using the micro Raman system, Jobin Yvon Horiba LABRAM-HR visible (400–1100 nm), with argon (488 nm) as the excitation source. Scanning electron microscopy images and EDAX spectrum were obtained using the SEM instrument model JEOL JSM-5600 with a resolution of 3.5 nm, magnification power of ×18–300 000 kV (in 136 steps), and acceleration voltage of 0.5–30 kV (53 steps) and energy-dispersive spectrometer, model INCA Oxford. The temperature dependence of resistivity for the sample La_0.67_Sr_0.33_MnO_3_ has been examined using the conventional dc four-probe method in the temperature range of 5–350 K. In addition, the differential scanning calorimetry (DSC) heat capacity measurement was carried out for below and above room temperature range keeping in view the temperature for the possible phase transition.

## 3. Results and discussions

### 3.1. Structural analysis

**Fig. 1:**
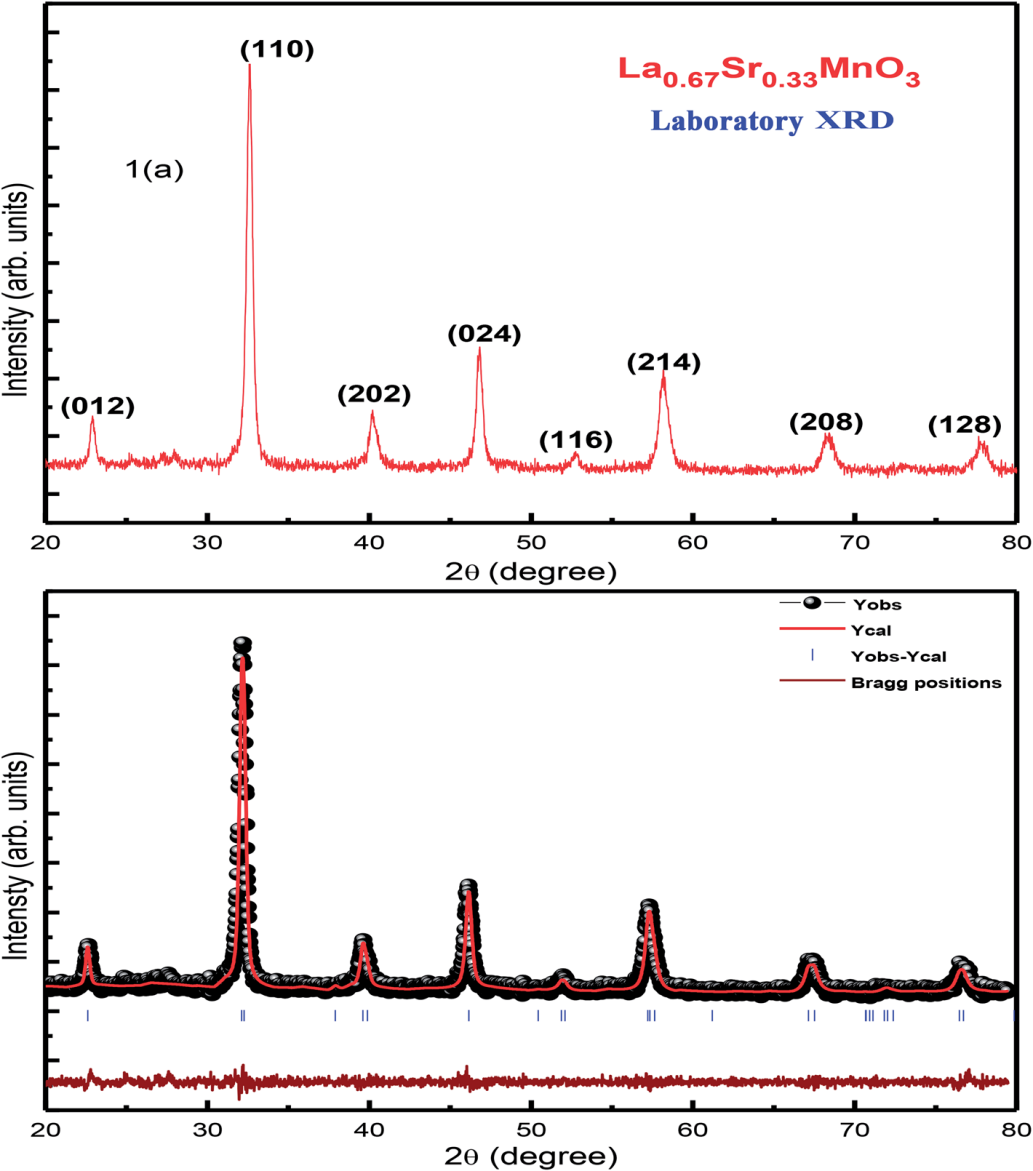
(a) RT-XRD spectrum of La_0.67_Sr_0.33_MnO_3_ (LSMO) and (b) Rietveld refinement of the XRD data of LSMO.

XRD characterization was carried out to investigate the phase formation and probe the structure of the synthesized La_0.67_Sr_0.33_MnO_3_. The analysis of room-temperature XRD pattern of the La_0.67_Sr_0.33_MnO_3_ sample shown in Fig. 1(a) reveals that a pure and single-phase perovskite structure has been successfully formed. No trace of secondary phase was detected within the sensitivity limit of the experiment. All the patterns were in good accordance with the JCPDS card no. 01-075-0440.

The XRD pattern has been indexed by a rhombohedral (hexagonal setting) lattice with the space group *R*3*c*. The broadness with a large full width at half maximum (FWHM) of characteristic XRD peak infers the formation of nanocrystals with the average crystallite size of the order of ≈19 nm. This is calculated using the Scherrer formula *D* = *kλ*/[*β* cos *θ*], where *D* is the average crystallite size, *λ* is the wavelength of X-ray used (1.5406 Å), *k* is a constant (shape factor ≈ 0.9), *θ* is the angle of diffraction, and *β* is the FWHM.^19^ In addition, the micro-strain, (*ε* = *β*/4 tan *θ*) = 0.38, and dislocation density, (*δ* = 1/*D*^2^) = 2.7564 calculated as *δ* × 10^15^ (lines per m^2^), were determined manually.

**Fig. 2:**
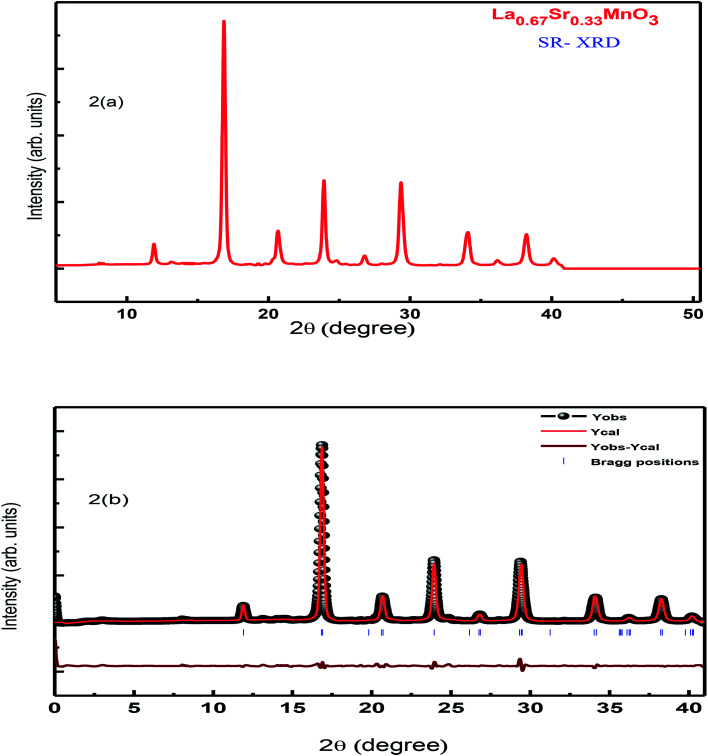
(a) SR-XRD of LSMO and (b) Rietveld refinement of the SR-XRD pattern of LSMO.

Further investigation on the structure and any of the phase transition of the sample was carried out using SR-XRD. Fig. 2(a) shows the SR-XRD pattern conforming the structure of the synthesized La_0.67_Sr_0.33_MnO_3_ sample, and the calculation of the crystallite size reveals the reduced value ≈ 16 nm as compared to the calculated crystallite size of the sample from the room temperature laboratory XRD using the Scherrer formula; this may be possible due to the noisy data obtained from RT-XRD and lesser effectiveness as compared to that of the SR-XRD as SR-XRD has higher brightness and intensity, high collimation, low emittance, higher polarization, and monochromatization, and for superfast resolved studies, it inherits pulsed light emission.^20^

Rietveld refinement of the RT-XRD pattern is shown in Fig. 1(b), which reveals the structure, lattice parameters, density, and cell volume, and all other parameters given in Table 1, which are consistent with the reported results.^21–23^ Moreover, the SR-XRD data have been Rietveld refined, as shown in Fig. 2(b), and the various parameters are shown in Table 2. The structural parameters are clearly reduced; this may be attributed to the intrinsic features of the SR-XRD as compared to those of the RT-XRD as synchrotron radiation is inherently advantageous as compared to laboratory XRD for several reasons. Among these reasons, the most important are its high brightness and high intensity with many orders of magnitude greater than those of XRD, high collimation, high-degree polarization, low emittance, which means that the product of source cross-section and solid angle of emission is small, large tunability in wavelength by monochromatization, and pulsed light emission that allows ultra-fast time-resolved studies.

**Table 1:** Details of Rietveld refined RT-XRD pattern of the La_0.67_Sr_0.33_MnO_3_

Parameters		Values obtained
Space group		*R*3*c*
*a* (Å)		5.573(3)
*c* (Å)		13.551(3)
*V* (Å^3^)		364.431
Density (g cm^−3^)		6.227
La(*x*, *y*, *z*)		(0.0, 0.0, 0.25)
Sr(*x*, *y*, *z*)		(0.0, 0.0, 0.25
Mn(*x*, *y*, *z*)		(0.0, 0.0, 0.0)
O(*x*, *y*, *z*)		(−0.448(3), 0.0, 0.25)
Bond distance	La/Sr–O	2.79(3) Å
	Mn–O	1.98(3) Å
*R*_F_		3.12
*R*_Bragg_		3.39
*R*_wp_		22.9
*R*_exp_		21.7
*R*_p_		26.8
*χ* ^2^		1.111
GOF		1.1

**Table 2:** Details of Rietveld refined SR-XRD pattern of the La_0.67_Sr_0.33_MnO_3_

Parameters		Values obtained
Space group		*R*3*c*
*a* (Å)		5.448(3)
*c* (Å)		13.425(3)
*V* (Å^3^)		345.0648
Density (g cm^−3^)		6.444
La(*x*, *y*, *z*)		(0.0, 0.0, 0.25)
Sr(*x*, *y*, *z*)		(0.0, 0.0, 0.25)
Mn(*x*, *y*, *z*)		(0.0, 0.0, 0.0)
O(*x*, *y*, *z*)		(−0.478(3), 0.0, 0.25)
Bond distance	La/Sr–O	2.74(3) Å
	Mn–O	1.93(2) Å
*R*_F_		0.850
*R*_Bragg_		0.987
*R*_wp_		9.51
*R*_exp_		4.38
*R*_p_		7.2
*χ* ^2^		3.5
GOF		1.9

**Fig. 3:**
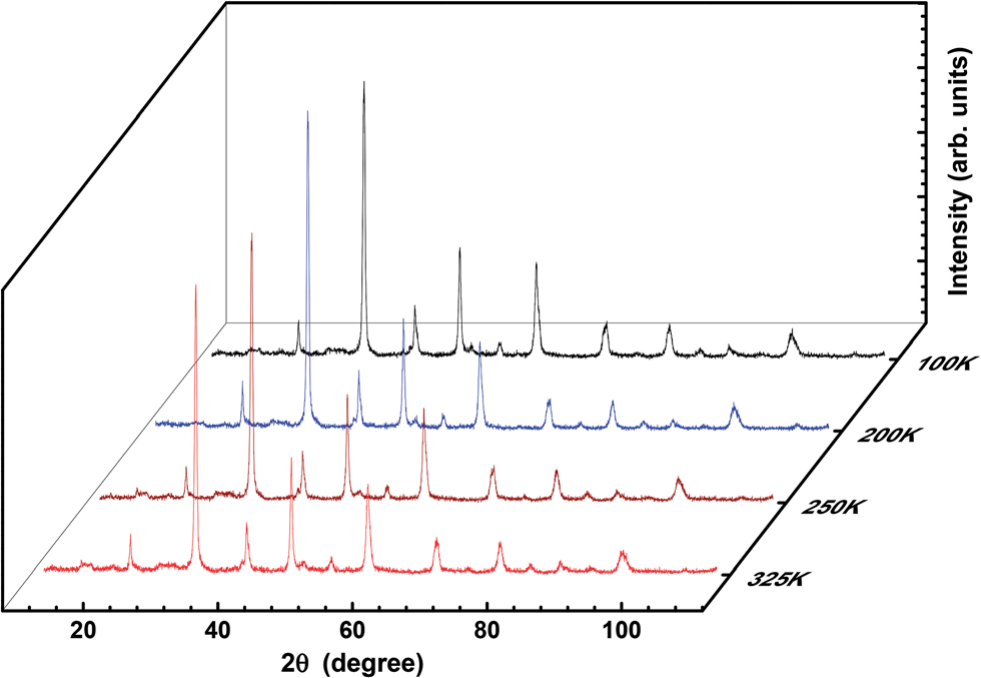
3D plot of temperature-dependent XRD of LSMO.

To verify any phase transition, we further performed low-temperature X-ray diffraction (LT-XRD). Fig. 3 depicts the XRD spectrum obtained at the temperatures 100 K, 200 K, 250 K, and 325 K with extended Bragg angular range, *i.e.*, 2*θ* (10° < 2*θ* < 110°). No new peaks were detected within the limits of the experiment carried out herein; this confirms that the prepared sample is single phased and highly pure. However, the intensity and the sharpness of the peaks at low temperatures are slightly sharper than the reflections at higher temperatures. In addition, there is hardly any shift in the peaks with variation in temperature; this denies any phase transition and reveals the single-phase nature of the crystals prepared herein. The analysis and comparison of the results obtained from the LT-XRD with the abovementioned results revealed their high agreement, which inferred that the synthesized sample was pure, single phased, and crystalline in nature. The increase in intensity obtained by lowering the temperature of the experiment enables the collection of the valid data as the lattice vibrations interfering in the actual reflections are arrested; this in turn improves the peak intensity to the background ratio *i.e.*, in background, extra thermal contributions get minimized; hence, the peak intensity gets enhanced.^24,25^

**Fig. 4:**
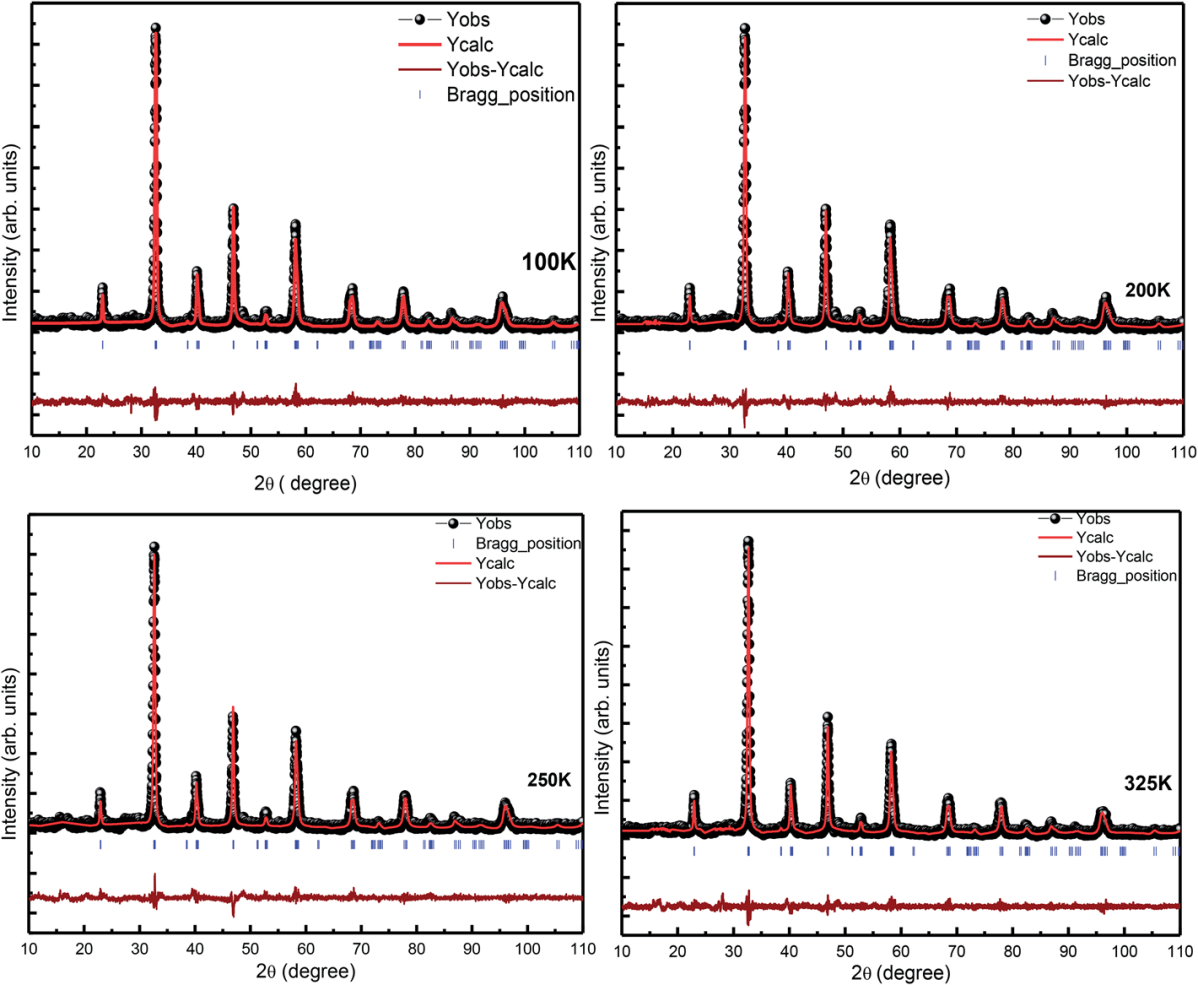
Rietveld refinement of the temperature-dependent XRD pattern of LSMO.

**Fig. 5:**
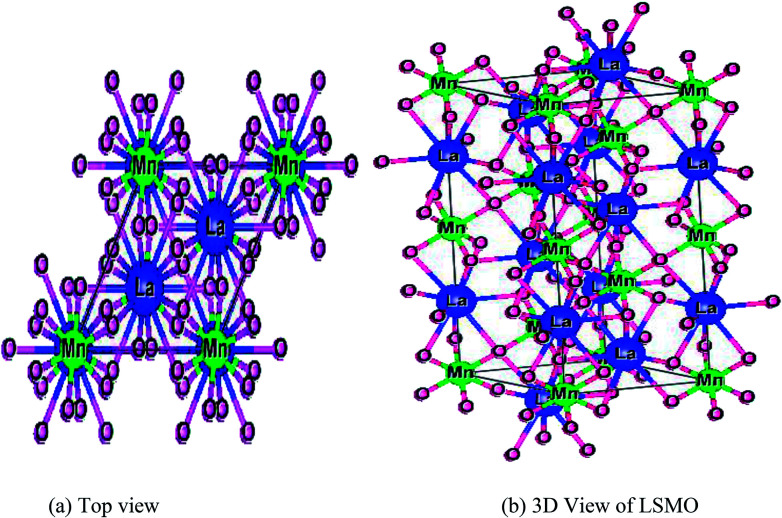
(a) Top view and (b) 3-dimensional structure of the prepared LSMO sample.

For further confirmation of the phase purity, Rietveld refinement has been conducted on the data represented in Fig. 3, and a slight variation in certain variables, which although is negligible, may be attributed to the thermal effects. The refinements are achieved; hence, the detailed data are shown in Fig. 4 and illustrated in Table 3. The structure of the prepared sample is shown in Fig. 5 that clearly represents the MnO_6_ octahedra.

**Table 3:** Details of Rietveld refinements of temperature-dependent XRD patterns of the La_0.67_Sr_0.33_MnO_3_

Parameters		Values obtained at different temperatures			
		100 K	200 K	250 K	325 K
Space group		*R*3*c*	*R*3*c*	*R*3*c*	*R*3*c*
*a* (Å)		5.503(3)	5.486(3)	5.494(3)	5.494(3)
*c* (Å)		13.369(3)	13.330(3)	13.360(3)	13.360(3)
*V* (Å^3^)		350.587	347.447	349.964	349.2429
Density (g cm^−3^)		6.805	6.771	6.788	6.712
La(*x*, *y*, *z*)		(0.0, 0.0, 0.25)	(0.0, 0.0, 0.25)	(0.0, 0.0, 0.25)	(0.0, 0.0, 0.25)
Sr(*x*, *y*, *z*)		(0.0, 0.0, 0.25)	(0.0, 0.0, 0.25)	(0.0, 0.0, 0.25)	(0.0, 0.0, 0.25)
Mn(*x*, *y*, *z*)		(0.0, 0.0, 0.0)	(0.0, 0.0, 0.0)	(0.0, 0.0, 0.0)	(0.0, 0.0, 0.0)
O(*x*, *y*, *z*)		(−0.463(3), 0.0, 0.25)	(−0.458(3), 0.0, 0.25)	(−0.465(3), 0.0, 0.25)	(−0.4578(3), 0.0, 0.25)
Bond distance	La/Sr–O	2.74(3) Å	2.74(3) Å	2.75(3) Å	2.744(3) Å
	Mn–O	1.95(3) Å	1.9(5) Å	1.95(3) Å	1.95(3) Å
*R*_F_		5.55	4.68	5.41	4.08
*R*_Bragg_		6.66	6.21	6.27	4.26
*R*_wp_		24.9	24.5	26.4	24.3
*R*_exp_		21.2	21.5	21.4	21.7
*R*_p_		23.5	23.6	24.8	23.0
*χ* ^2^		1.39	1.34	1.52	1.26
GOF		1.2	1.2	1.2	1.1

### 3.2. Raman scattering measurements

**Fig. 6:**
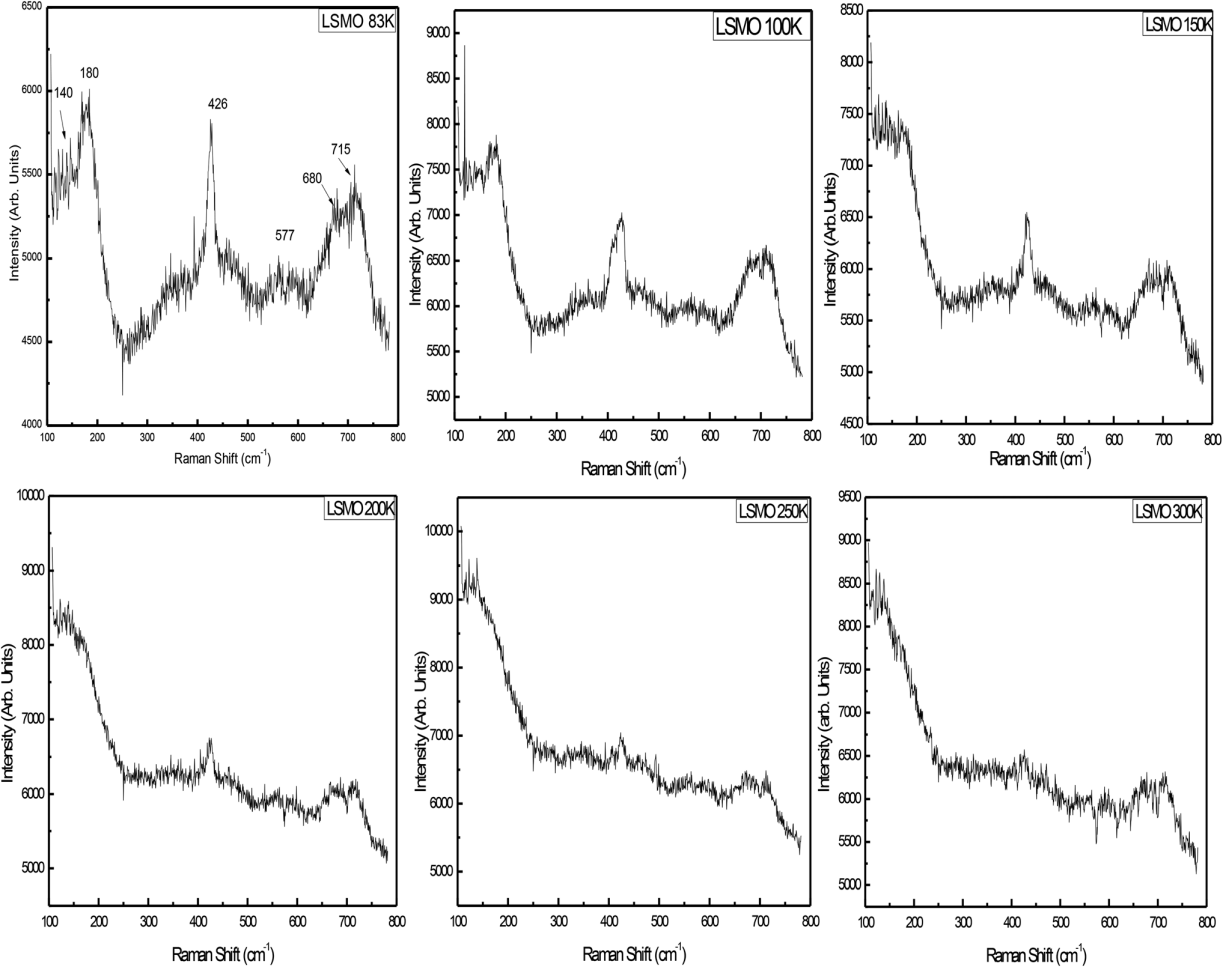
Temperature-dependent Raman spectra of LSMO.

Fig. 6 displays the room-temperature Raman spectra of the as-prepared La_0.67_Sr_0.33_MnO_3_ nanopowder. For an ideal cubic perovskite structure of LaMnO_3_, no phonon mode is Raman active, and it is the orthorhombic or rhombohedral distortion that gives rise to Raman-active phonon modes. The intensity and width of these modes represent deviation from the ideal cubic structure, and in turn, these bands represent contribution of the Jahn–Teller (JT) effect in the system. The Raman spectrum of the ferromagnetic metallic (*Pbnm*) phase or rhombohedral (*R*3*c*) La_0.67_Sr_0.33_MnO_3_ having no stretching phonon modes belongs to the compounds that do not possess JT distortion. The Jahn–Teller distortion decreases as a result of the introduction of Mn^4+^ ions by the higher doping of Sr; this in turn leads to the regular structure and retain the tilt of the octahedra. For a higher Sr doping or for oxygen-deficient samples, the structure has been reported to be rhombohedral with tilted octahedra and strictly identical Mn–O bonds.^26,27^

There are thirty vibrational degrees of freedom for the rhombohedral structure: *Γ*_vib_ ≡ 2A_1u_ + 3A_2g_ + A_1g_ + 4A_2u_ + 4E_g_ + 6E_u_. Among these, 1A_g_ + 4E_g_ are the Raman-active modes, 3A_2u_ + 5E_u_ are IR active, and the remaining 2A_1u_ + 3A_2g_ are silent modes. For this structure, the Raman-active modes can be classified into 1A_1g_ + 1E_g_ rotational or tilt modes, 1E_g_ bending, and 1E_g_ anti-stretching of the MnO_6_ octahedra, and the remaining E_g_ is related to the vibration of A ions. The symmetric stretching mode is A_2g_ and therefore not observable.^28^

Raman scattering experiments down to liquid nitrogen temperature were performed on the La_0.67_Sr_0.33_MnO_3_ single crystal, and the data analysis indicated that the mode at 180 cm^−1^ was an A_1g_ symmetry mode associated with MnO_6_ octahedra, an out-of-phase rotation, and the mode observed at 426 cm^−1^ in a single crystal is assigned as an E_g_ symmetry mode linked to an internal mode (bending) of the MnO_6_ octahedra.^17,29^ The signature peak at 140 cm^−1^ followed by the peaks at 180 and 426 cm^−1^ can be considered as a characteristic of the diminished Jahn–Teller distortion. The metallic state is reflected in the Raman spectrum as a total reduction of the JT-distortion-induced bands along with the appearance of these peaks,^27,30^ and these Raman bands attain strength as the temperature is reduced. Broadening of the JT-induced peaks with higher Sr in LaMnO_3_ may be attributed to the site disorder-induced effects believed to exist due to higher excitation source and may be attributed to the resonance effect.^31^

The lattice dynamics calculations have predicted a breathing mode with A_2g_ symmetry on LaMnO_3_, the rhombohedrally distorted structure at about 716 cm^−1^, which is the silent mode.^32^ The mode observed at 715 cm^−1^ in La_0.67_Sr_0.33_MnO_3_ could be assigned as being an A_2g_ symmetry mode. It is argued that the mode at 715 cm^−1^ can be possibly assigned to the phonon density of states feature rather than to the A_2g_ silent mode.^28,32^ Supported by the model proposed by Iliev *et al.*, the diminished and lower side-shifted (because of higher excitation source) modes around 550 cm^−1^ are assigned as a density of states feature activated by the loss of local symmetry in the O (oxygen) sub-lattice.^30^ The band at 680 cm^−1^ cannot be assigned to the manganite as its intensity varies from measurement to measurement, but may be attributed to the small volume of manganese oxides as compared to Mn_3_O_4_ that has a strong reflection about this frequency.^33^

### 3.3. Morphology studies

**Fig. 7:**
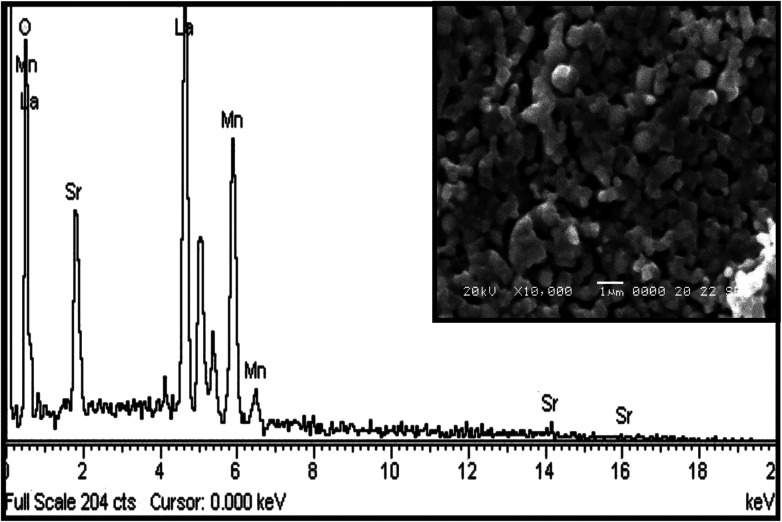
EDAX spectrum of LSMO with an inset showing the SEM image.

The energy-dispersive X-ray analysis (EDAX) shows that there is no new element in the sample under study; hence, the composition of the compound is confirmed within the experimental limits. Fig. 7 represents the EDX spectra of La_0.67_Sr_0.33_MnO_3_, which confirms that all integrated elements, *i.e.*, La, Sr, Mn, and O, are present and reveals that there is no loss during the sintering. The SEM images shown in the inset of Fig. 7 reveal that the sample sintered at 1150 °C comprises homogeneous particles that do not connect with each other tightly and larger grains seem to be well separated by smaller grains. The grains of the sample have excellent crystallinity and show agglomeration in ellipsoidal shapes with the average particle sizes of 250 nm or 0.25 μm determined using the ImageJ software. It is obvious that at a higher sintering temperature of 1150 °C, the average particle size of the sample increases as compared to the average particle size obtained from the powder XRD spectrum after calcination at 800 °C. As is well known, a higher sintering temperature facilitates diffusion of crystallites or grains resulting in the growth of the grain size. Since the particle holds more than one grain or crystallite, the average particle size increases and leads to the reduction of the strain as compared to a smaller grain size; this is well revealed by the results.^34^

### 3.4. dc resistivity

**Fig. 8:**
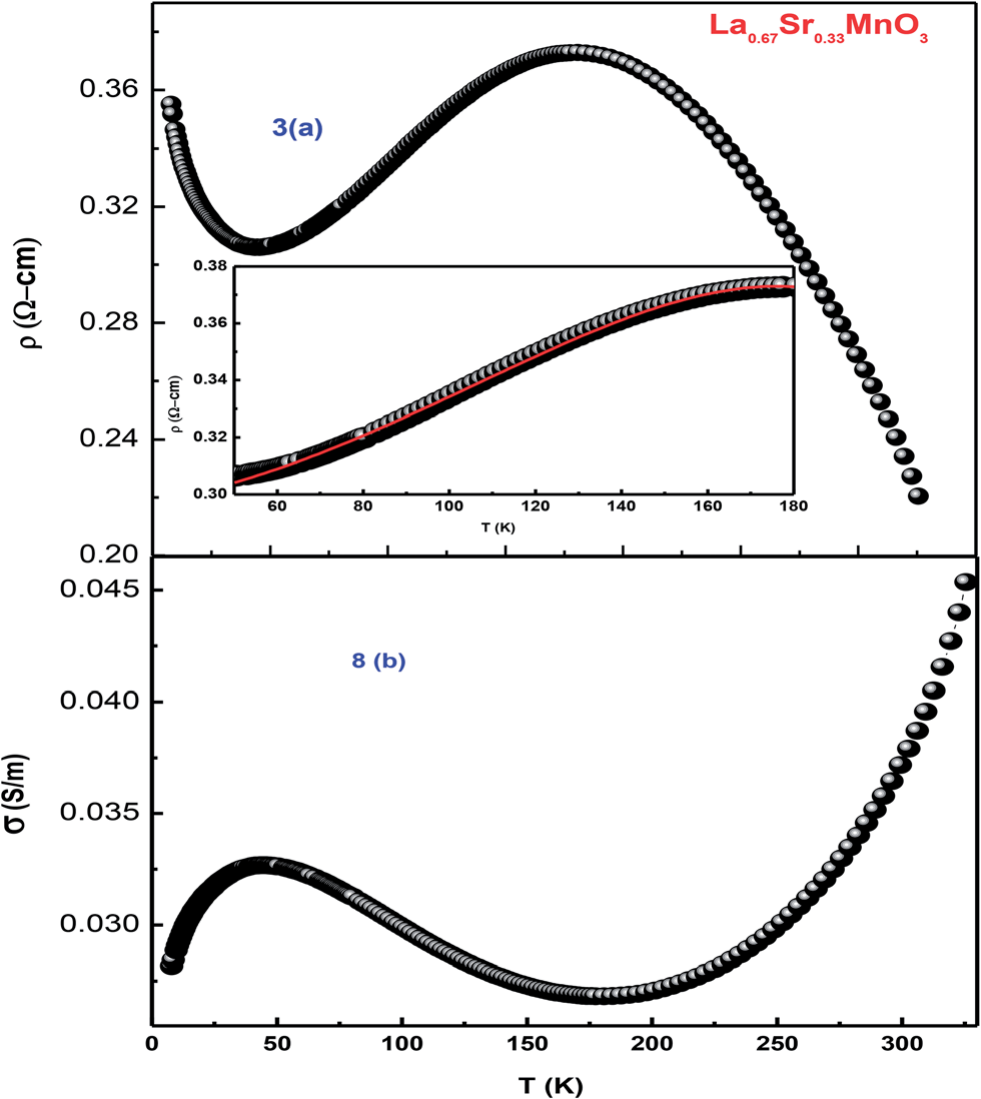
(a) Temperature-dependent resistivity of LSMO and (b) conductivity as a function of temperature.

As the sample La_0.67_Sr_0.33_MnO_3_ is metallic in nature, the resistance is very small, which is effectively measured by a conventional four-probe technique to eliminate the contact resistance between the sample and electrical contacts. The measurement was carried out by passing a constant current through one pair of electrical leads on the extremes of the rectangular bar-shaped sample and measuring the potential due to the resistance of the specimen by another pair of leads. As the measurement is susceptible to thermal gradients, the thermo-emfs are eliminated by measuring voltages in both directions of currents by reversal of sign of the current and subtracting them as *V*^+^ = *V* + *V*_th_, *V*^−^ = −*V* − *V*_th_, *V* = (*V*^+^ − *V*^−^)/2, where *V*^+^ is the voltage developed for the current in one direction, *V*^−^ is the voltage developed for the other direction, and *V*_th_ is the voltage that comes into existence due to the thermal gradient. For the measurement, the sample has been cut in the form of a rectangular bar with the dimensions of *l* = 1.2 mm, *t* = 1.6 mm, and *b* = 4.8 mm. Fig. 8[(a) and (b)] show the variation of electrical resistivity as well as conductivity of La_0.67_Sr_0.33_MnO_3_ as functions of temperature with the applied current of 50 μA. The metal-insulator transition temperature (*T*_MI_) was found to be 178 K. The lower value of *T*_MI_ is due to a lower grain size, and the result is supported by the literature.^21^ The *T*_MI_ value is far below the corresponding *T*_C_ value, associated with an apparent increase in resistivity. To this point, the reason for large discrepancy between *T*_MI_ and *T*_C_ is not known, and it has been stated that this huge discrepancy in nanostructured manganites is a mystery that requires a serious attention. As electrical transport properties depend not only on the size of the grains of the samples but also on the porosity of the pellets, it is found that the sample calcined and sintered at higher temperatures attains a larger grain size and displays an appreciable decrease in resistivity. This characteristic is mainly attributed to the reduction in porosity with an increase in the sintering temperature. Since in our study, we sintered our sample at 1150 °C that resulted in the increased grain size and reduced porosity, the result was a sharp decrease in *T*_MI_ consistent with the earlier observation.^35^

An insight can be gained about the witnessed transport behavior in manganites using the phenomenological Core–Shell Model (CSM)^35^ based on spin-polarized tunnelling (SPT) according to which the blocking temperature (*T*_B_ < *T*_C_) is decided by magnetic exchange energy, anisotropy energy, and thermal energy, which are strongly competitive in nature. The SPT of conduction electrons appears in the low-temperature FM region, where the blocked state of core moments exists that creases the metallic state due to which a gradual drop in *T*_B_ is natural with a decrease in grain size; this in turn results in the reduction of *T*_MI_. In addition, the sample shows resistivity minima below 48 K due to charge-carrier tunnelling between AFM-coupled grains.^36^ La_0.67_Sr_0.33_MnO_3_ exhibits metallic nature below its *T*_MI_, and the conduction mechanism in the regime is understood by fitting the measured data using a well-known equation^37^

1 *ρ* = *ρ*_0_+ *ρ*_2_*T*^2^ + *ρ*_4.5_*T*^4.5^

where *ρ*_0_, *ρ*_2_, and *ρ*_4.5_ are the resistivity arising due to grain/domain boundary scattering, electron–electron scattering, and mixed effects of electron–electron, electron–magnon, and electron–phonon scattering processes, respectively. The best fit to eqn (1) is shown in the inset of Fig. 8(a). The values obtained infer that in La_0.67_Sr_0.33_MnO_3_, electron–electron scattering plays a dominant role in the conduction mechanism in the metallic region.

**Fig. 9:**
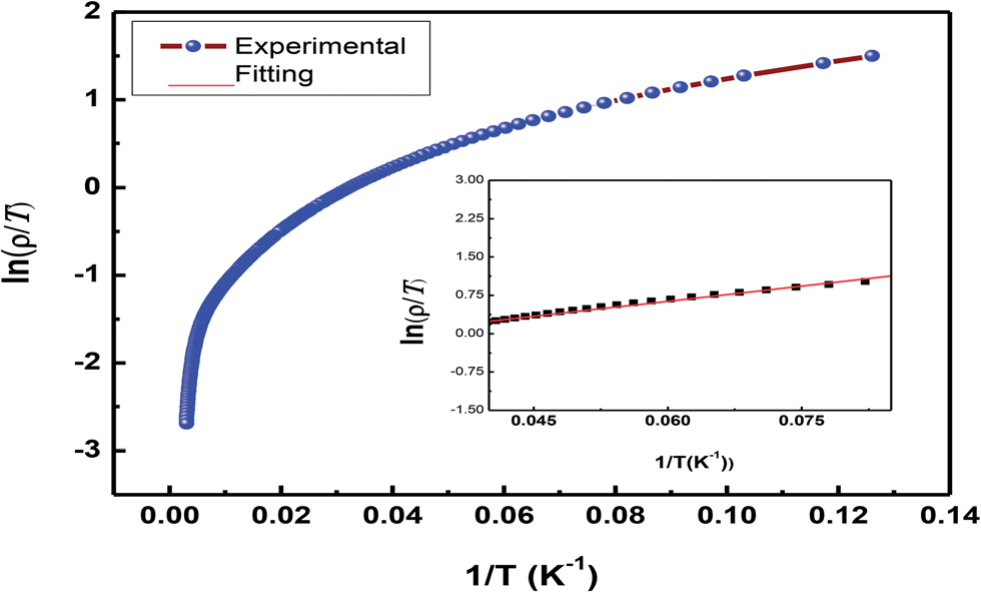
Plot of ln(*ρ*/*T*) *vs.* 1/*T* representing the SPH model.

To demonstrate the charge transport mechanism, ln(*ρ*/*T*) has been plotted in the high-temperature region as a function of 1/*T* in Fig. 9. The good linear fit shown in the inset in the same figure using the equation *ρ* = *ρ*_0_*T* exp(*E*_a_/*K*_B_*T*), where *ρ* is the resistivity, *T* is the absolute temperature, and *E*_a_ denotes the activation energy, shows conduction in the paramagnetic semiconducting region, which reveals that the sample obeys the small polaron hopping model (SPH). The value of *E*_a_ ≈ 1.7 meV, which is of the very low order and attributed to a smaller particle size and a good conducting nature of the sample, as has been discussed above.

**Fig. 10:**
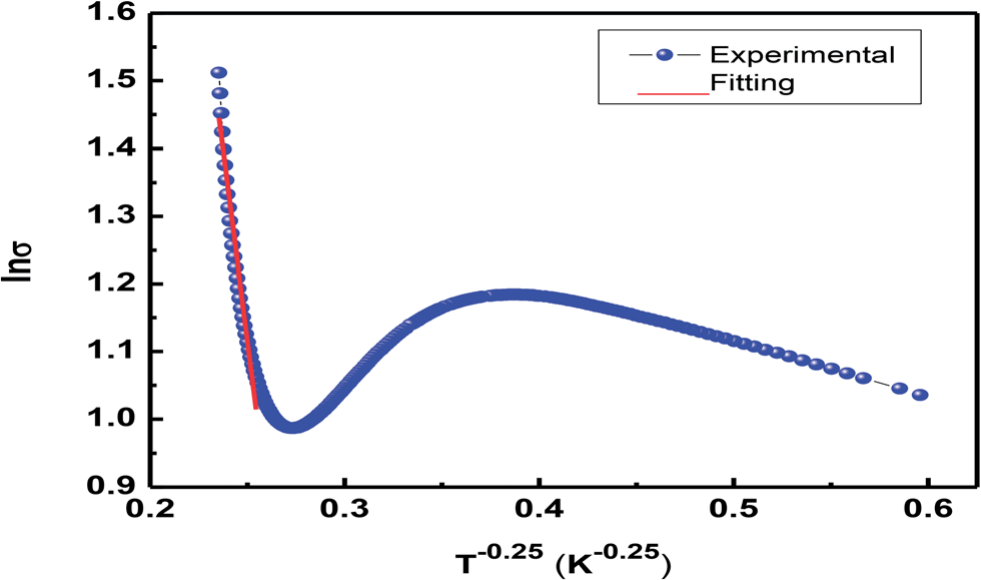
Plot of ln *σ vs. T*^−1/4^ to present the VRH model.

The expression *ρ* ≈ *ρ*_0_ exp(*T*/*T*_0_)^1/4^ represents resistivity in the variable range hopping (VRH) model, where *ρ*_0_ is speculated to be dependent on electron–phonon interaction, and in most of the cases, it is taken as a constant despite the fact that there is very small impact of the temperature; *T*_0_ is the characteristic temperature of the material that depends on Mott localization energy and is given by the expression *T*_0_ = [*λα*^3^]/[*k*_B_*N*(*E*_F_)], where *λ* (≈18) is a dimensionless constant, *N*(*E*_F_) is the density of states, and *α* represents inverse localization length (1/*ζ*) taken as 2.22 nm^−1^ for calculations.^38^Fig. 10 shows variations in ln *σ vs. T*^−1/4^. The density of states was found to be of a higher order than that of usual oxide semiconductors. The higher value of *N*(*E*_F_) is believed to exist due to the influence of the adiabatic small polaron hopping process.^39,40^ The value of *N*(*E*_F_) ≈ 8.69 × 10^−24^ eV cm^−3^ calculated herein is in good agreement with the reported data.^38–40^ The observed high value of *N*(*E*_F_) validates the small polaron hopping nature of the carriers in the manganite under investigation.

### 3.5. Heat capacity studies

Differential scanning calorimetry (DSC) is a thermo-analytical technique where the difference in the heat energy required for increasing the temperature of a sample and reference is measured as a function of temperature. It is necessary that the reference sample possesses well-defined heat capacity over the range of temperatures to be scanned. For phase transition in a sample, less or more heat needs to flow to it as compared to the case of the reference such that both the samples are maintained at the same temperature. Specific heat measurement, *C*_P_(*T*), is a direct measure of the occurrence of thermodynamical phase transition. The total specific heat at low temperatures is given by the equation

**Fig. 11:**
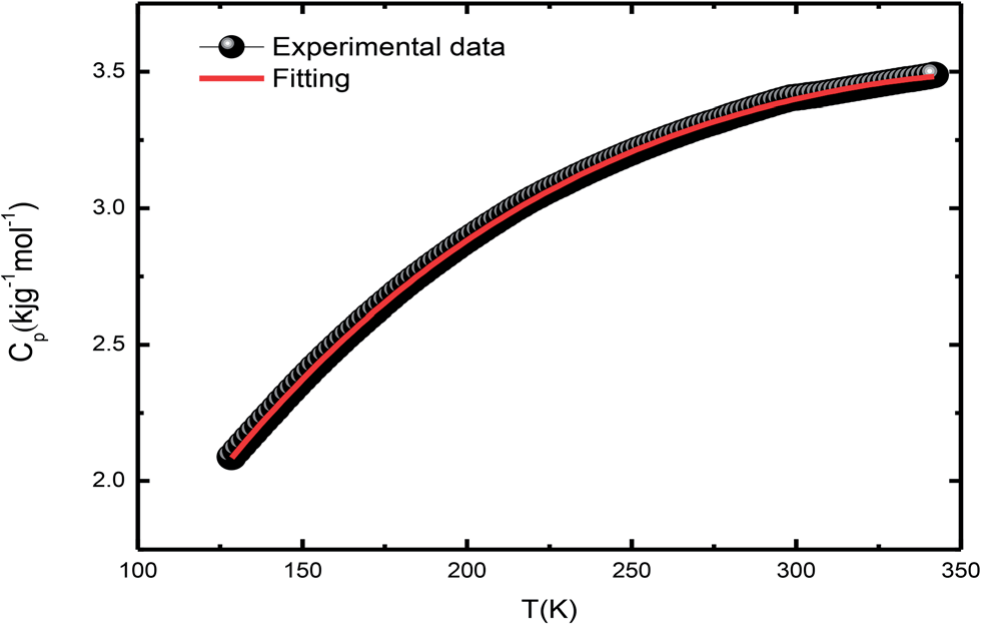
Plot for DSC-specific heat.

2 *C*_p_(total) = *C*_lat_ + *C*_el_ + *C*_mag_ + *C*_hyp_

herein, *C*_el_ = *γT* is the electronic contribution from free charge carriers, and *C*_hyp_ = *α*/*T*^2^ is the hyperfine contribution arising from the local magnetic field due to ^55^Mn nuclei due to the presence of electrons in unfilled shells. *C*_mag_ = *δT*^*n*^, where the value of exponent depends on the nature of magnetic excitation, namely *n* = 3/2 for ferromagnetic spin waves and *n* = 2 for antiferromagnetic spin waves. However, high-temperature-specific heat data of the LSMO single crystal displayed in Fig. 11 fitted using eqn (2) is the contribution of the two terms, and the abovementioned equation can be reduced to

3 *C*_p_(total) = *C*_lat_(*T*) + *C*_SW_(*T*)

herein, *C*_latt_ = *β*_3_*T*^3^ + *β*_5_*T*^5^ is the lattice contribution induced by the phonons, and *C*_SW_ = *β*_3/2_*T*^3/2^ is the magnetic contribution induced by the ferromagnetic spin waves. As is clear from the figure, the specific heat capacity smoothly increases below 295 K and then from 300 K onwards. In between these two temperatures, we have observed an anomaly that shows a light kink in the peak in the vicinity of *T* ≈ 298 K indicative of a possible phase transformation.

During the analysis, we considered all possible combinations to obtain the best fit condition. However, the agreement was poor in the extended temperature range. The addition of the *β*_5_*T*^5^ term in *C*_lat_ does not improve the fitting, but generates unphysical values in a limited temperature range. As La_0.67_Sr_0.33_MnO_3_ shows insulating behavior at low temperatures, as expected, *C*_el_ has little contribution in *C*_p_(total), *C*_hyp_ does not contribute to *C*_p_(total), and its incorporation results in a negative contribution and unphysical values for *C*_p_(total).^41,42^

## 4. Conclusions

The single-phase and crystalline nanopowder of La_0.67_Sr_0.33_MnO_3_ was successfully prepared by the sol–gel auto-combustion method by maintaining the pH at 11 using ammonia. Ethylene glycol (EG) addition effectively helped in controlling the particle size, and citric acid played the role of a chelating agent as well as an organic fuel in the combustion and calcination process. The crystal structure was verified by RT-XRD, SR-XRD, and Rietveld refinement and was found to exhibit a rhombohedral structure with the space group *R*3*c*. The purity of the sample is appreciable as no phase transition is observed in a wide range of temperature, *i.e.*, above and below room temperature. The temperature-dependent resistivity confirmed the transition temperature (*T*_MI_) ≈ 178 K. Temperature-dependent Raman characterization confirmed a metallic phase with a total reduction of J–T distortion in the rhombohedral LSMO indicated by the appearance of new peaks at 140 and 426 cm^−1^. The DSC specific heat measurement confirmed the ferromagnetic metallic nature and hence an increase in the heat capacity with an increase in temperature. EDAX confirmed the presence of all the constituents, and the SEM image confirmed the growth of the grain size as a result of sintering at high temperatures.

## Conflicts of interest

There are no conflicts to declare.

## Supplementary Material

## References

[cit1] Ramirez A. (1997). J. Phys.: Condens. Matter.

[cit2] Coey J., Viret M., Molnar S. V. (1999). Adv. Phys..

[cit3] Dagotto E., Hotta T., Moreo A. (2001). Phys. Rep..

[cit4] Helmholt R. V., Wecker J., Holzapfel B. (1993). Phys. Rev. Lett..

[cit5] Chahara K., Ohno T., Kasai M. (1993). Appl. Phys. Lett..

[cit6] Jin S., Tiefel T. H., McCormack M. (1994). Science.

[cit7] Goodenough J. B. (1955). Phys. Rev. B.

[cit8] Topfer J., Goodenough J. B. (1997). J. Solid State Chem..

[cit9] Murakami Y., Hill J. P., Gibbs D., Blume M., Koyama I., Tanaka M., Kawata H., Arima T., Tokura Y., Hirota K., Endoh Y. (1998). Phys. Rev. Lett..

[cit10] Kim M. W., Moon S. J., Jung J. H., Yu J., Parashar S., Murugavel P., Lee J. H., Noh T. W. (2006). Phys. Rev. Lett..

[cit11] Zener C. (1951). Phys. Rev..

[cit12] Shaikh M. W., Varshney D. (2012). Mater. Chem. Phys..

[cit13] Mansuri I., Varshney D., Kaurav N., Lu C. L., Kuo Y. K. (2011). J. Magn. Magn. Mater..

[cit14] Moriya T. (1960). Phys. Rev..

[cit15] Matsumoto G. (1970). J. Phys. Soc. Jpn..

[cit16] Chainani A., Mathew M., Sarma D. D. (1993). Phys. Rev. B.

[cit17] Urushibara A., Moritomo Y., Arima T., Asamitsu A., Kido G., Tokura Y. (1995). Phys. Rev. B.

[cit18] Rodríguez-Carvajal J. (1993). Phys. B.

[cit19] Mendelson M. I. (1969). J. Am. Ceram. Soc..

[cit20] Jordon-Sweet J. L. (2000). IBM J. Res. Dev..

[cit21] Lu W. J., Luo X., Hao C. Y., Song W. H., Sun Y. P. (2008). J. Appl. Phys..

[cit22] Reshmi C. P., Pillai S. S., Vasundhara M., Raji G. R., Suresh K. G., Varma M. R. (2013). J. Appl. Phys..

[cit23] Mnegfui S., Dhahri A., Dhahri J., Hill E. (2013). J. Supercond. Novel Magn..

[cit24] Goeta E., Howard J. A. K. (2004). Chem. Soc. Rev..

[cit25] Mishra S. K., Pandey D. (2009). Appl. Phys. Lett..

[cit26] Rodríguez-Carvajal J., Hennion M., Moussa F., Moudden A. H., Pinsard L., Revcolevschi A. (1998). Phys. Rev. B.

[cit27] Sathe V. G., Rawat R., Dubey A., Narlikar A. V., Prabhakaran D. (2009). J. Phys.: Condens. Matter.

[cit28] Abrashev M. V., Litvinchuk A. P., Iliev M. N., Meng R. L., Popov V. N., Ivanov V. G., Chakalov R. A., Thomsen C. (1999). Phys. Rev. B.

[cit29] Granado E., Moreno N. O., Garcma A., Sanjurjo J. A., Rettori C., Torriani I., Oseroff S. B., Neumeier J. J., McClellan K. J., Cheong S. W., Tokura Y. (1998). Phys. Rev. B.

[cit30] Iliev M. N., Abrashev M. V., Popov V. N., Hadjiev V. G. (2003). Phys. Rev. B.

[cit31] Kruger R., Schulz B., Naler S., Rauer R., Budelmann D., Backstrom J., Kim K. H., Cheong S. W., Perebeinos V., Rubhausen M. (2004). Phys. Rev. Lett..

[cit32] Souza Filho G., Faria J. L. B., Guedes I., Sasaki J. M., Freire P. T. C., Freire V. N., Mendes Filho J., Xavier M. M., Cabral F. A. O., de Araújo J. H., da Costa J. A. P. (2003). Phys. Rev. B.

[cit33] Liu X., Xu S., Kato K., Moritomo Y. (2002). J. Phys. Soc. Jpn..

[cit34] Siwach P. K., Prasad R., Gaur A., Singh H. K., Varma G. D., Srivastava O. N. (2007). J. Alloys Compd..

[cit35] Dey P., Nath T. K. (2006). Phys. Rev. B.

[cit36] Rozenberg E., Auslender M., Felner I., Gorodetsky G. (2000). J. Appl. Phys..

[cit37] Dar M. A., Varshney D. (2015). Solid State Commun..

[cit38] Alexandrov A. S. , and Mott N. F., Polarons and Bipolarons, World Scientific, Singapore, 1995

[cit39] Graziosi P., Gambardella A., Prezioso M., Riminucci A., Bergenti I., Homonnay N., Schmidt G., Pullini D., Mataix D. B. (2014). Phys. Rev. B.

[cit40] Jung W. H. (1998). J. Mater. Sci. Lett..

[cit41] Manna K., Elizabeth S., Kumar P. S. A. (2016). J. Appl. Phys..

[cit42] Tanaka J., Mirsushahi T. (1984). J. Phys. Soc. Jpn..

